# Supplementary material for: Associations of obesity-related indices with mild cognitive impairment in adults 60 years and older with type 2 diabetes: a retrospective study
Source: PeerJ. 2025 May 13;13:e19442. doi: 10.7717/peerj.19442 (PMC12083468; doi:10.7717/peerj.19442)
Supplement: Supplemental Information 2 [file peerj-13-19442-s002.docx]

| **Table S1** Detailed ROC curve statistics of the twelve obesity-related indices for identifying MCI among elderly patients with T2D | | | |
| --- | --- | --- | --- |
| Variable | AUC | Sensitivity | Specificity |
| BMI | 0.560 | 0.525 | 0.592 |
| WHR | 0.554 | 0.667 | 0.457 |
| WHtR | 0.575 | 0.580 | 0.545 |
| LAP | 0.669 | 0.676 | 0.641 |
| BRI | 0.575 | 0.594 | 0.543 |
| C-index | 0.556 | 0.703 | 0.394 |
| VAI | 0.679 | 0.712 | 0.601 |
| BAI | 0.547 | 0.530 | 0.576 |
| AVI | 0.580 | 0.365 | 0.766 |
| ABSI | 0.536 | 0.612 | 0.457 |
| TyG index | 0.673 | 0.653 | 0.633 |
| CMI | 0.682 | 0.849 | 0.457 |
