# Supplementary material for: Associations of obesity-related indices with mild cognitive impairment in adults 60 years and older with type 2 diabetes: a retrospective study
Source: PeerJ. 2025 May 13;13:e19442. doi: 10.7717/peerj.19442 (PMC12083468; doi:10.7717/peerj.19442)
Supplement: Supplemental Information 3 [file peerj-13-19442-s003.doc]

STROBE Statement—Checklist of items that should be included in reports of ***case-control studies***

|  | Item No | Recommendation |
| --- | --- | --- |
| **Title and abstract** | 1 | (*a*) Indicate the study’s design with a commonly used term in the title or the abstract  **Address: Page 2 Line 2** |
|  |  | (*b*) Provide in the abstract an informative and balanced summary of what was done and what was found  **Address: Page 2 Line 11-33** |
| Introduction | | |
| Background/rationale | 2 | Explain the scientific background and rationale for the investigation being reported  **Address: Page 3-4 Line 1-34** |
| Objectives | 3 | State specific objectives, including any prespecified hypotheses  **Address: Page 4 Line 34-37** |
| Methods | | |
| Study design | 4 | Present key elements of study design early in the paper  **Address: Page 5-6 Line 55-88.** |
| Setting | 5 | Describe the setting, locations, and relevant dates, including periods of recruitment, exposure, follow-up, and data collection  **Address: Page 4 Line 39-50; Page 5-6 Line 55-88.** |
| Participants | 6 | (*a*) Give the eligibility criteria, and the sources and methods of case ascertainment and control selection. Give the rationale for the choice of cases and controls  **Address: Page 4 Line 39-50.** |
|  |  | (*b*) For matched studies, give matching criteria and the number of controls per case  No matched studies. |
| Variables | 7 | Clearly define all outcomes, exposures, predictors, potential confounders, and effect modifiers. Give diagnostic criteria, if applicable  **Page 5-6 Line 55-88.** |
| Data sources/ measurement | 8* | For each variable of interest, give sources of data and details of methods of assessment (measurement). Describe comparability of assessment methods if there is more than one group  **Page 5-6 Line 55-88.** |
| Bias | 9 | Describe any efforts to address potential sources of bias  **Page 6 Line 89-104.** |
| Study size | 10 | Explain how the study size was arrived at  **Page 4 Line 39-50.** |
| Quantitative variables | 11 | Explain how quantitative variables were handled in the analyses. If applicable, describe which groupings were chosen and why  **Page 6 Line 89-104.** |
| Statistical methods | 12 | 1. Describe all statistical methods, including those used to control for confounding   **Page 6 Line 89-104.** |
|  |  | 1. Describe any methods used to examine subgroups and interactions   **Page 6 Line 89-104.** |
|  |  | 1. Explain how missing data were addressed   **Page 6 Line 89-104.** |
|  |  | 1. If applicable, explain how matching of cases and controls was addressed   **Page 6 Line 89-104.** |
|  |  | 1. Describe any sensitivity analyses   **Page 6 Line 89-104.** |
| Results | | |
| Participants | 13* | 1. Report numbers of individuals at each stage of study—eg numbers potentially eligible, examined for eligibility, confirmed eligible, included in the study, completing follow-up, and analysed   **Page 7 Line 106-108.** |
|  |  | (b) Give reasons for non-participation at each stage  **Figure 1** |
|  |  | 1. Consider use of a flow diagram   **Figure 1** |
| Descriptive data | 14* | 1. Give characteristics of study participants (eg demographic, clinical, social) and information on exposures and potential confounders   **Page 7 Line 108-114. Table 1** |
|  |  | 1. Indicate number of participants with missing data for each variable of interest   No missing data |
| Outcome data | 15* | Report numbers in each exposure category, or summary measures of exposure  **Page 7 Line 108-114. Table 1** |
| Main results | 16 | 1. Give unadjusted estimates and, if applicable, confounder-adjusted estimates and their precision (eg, 95% confidence interval). Make clear which confounders were adjusted for and why they were included   **Page 7-8 Line 115-154.** |
|  |  | 1. Report category boundaries when continuous variables were categorized   **Page 7-8 Line 115-154.** |
|  |  | (*c*) If relevant, consider translating estimates of relative risk into absolute risk for a meaningful time period |

| Other analyses | 17 | Report other analyses done—eg analyses of subgroups and interactions, and sensitivity analyses  **Page 7-8 Line 115-154.** |
| --- | --- | --- |
| Discussion | | |
| Key results | 18 | Summarise key results with reference to study objectives  **Page 8-9 Line 156-167.** |
| Limitations | 19 | Discuss limitations of the study, taking into account sources of potential bias or imprecision. Discuss both direction and magnitude of any potential bias  **Page 11-12 Line 238-245.** |
| Interpretation | 20 | Give a cautious overall interpretation of results considering objectives, limitations, multiplicity of analyses, results from similar studies, and other relevant evidence  **Page 12 Line 247-254.** |
| Generalisability | 21 | Discuss the generalisability (external validity) of the study results  **Page 12 Line 247-254.** |
| Other information | | |
| Funding | 22 | Give the source of funding and the role of the funders for the present study and, if applicable, for the original study on which the present article is based  **Page 1 Funding statement** |

*Give information separately for cases and controls.

**Note:** An Explanation and Elaboration article discusses each checklist item and gives methodological background and published examples of transparent reporting. The STROBE checklist is best used in conjunction with this article (freely available on the Web sites of PLoS Medicine at http://www.plosmedicine.org/, Annals of Internal Medicine at http://www.annals.org/, and Epidemiology at http://www.epidem.com/). Information on the STROBE Initiative is available at http://www.strobe-statement.org.
